# Treatment patterns and bleeding outcomes in persons with severe hemophilia A and B in a real-world setting

**DOI:** 10.1007/s00277-020-04250-9

**Published:** 2020-09-11

**Authors:** Cihan Ay, Leonard Perschy, Judit Rejtö, Alexandra Kaider, Ingrid Pabinger

**Affiliations:** 1 Clinical Division of Haematology and Haemostaseology, Department of Medicine I, Medical University of Vienna, Vienna, Austria; 2 Center for Medical Statistics, Informatics and Intelligent Systems, Medical University of Vienna, Vienna, Austria

**Keywords:** Hemophilia A, Hemophilia B, Factor VIII, Factor IX, Hemorrhage, Half-life

## Abstract

The current standard of care treatment for severe hemophilia A and B (SHA and SHB) is the prophylactic intravenous replacement of coagulation factor VIII or IX (FVIII/FIX) to prevent spontaneous bleeding. Persons with hemophilia without prophylactic treatment receive therapy in case of bleeding, i.e., on demand. To assess treatment patterns, utilization of products, and bleeding outcomes in a real-world cohort of persons with SHA and SHB, defined as FVIII or FIX activity < 1%, data was retrospectively collected from hemophilia-specific patient diaries used for home treatment, medical records, and entries into the Austrian Hemophilia Registry from the year 2012 to 2017. Fifty-three male persons with SHA (*n* = 47) and SHB (*n* = 6) were included; 26 with SHA and 5 with SHB were on prophylaxis, 8 and 1 switched therapy regimen, and 13 and 0 received on-demand therapy. Persons on prophylaxis used a mean factor FVIII or FIX dose of 71.7 and 40.1 IU/kg/week. Median (IQR) annualized bleeding rates (ABR) in SHA were 28.0 (23.4–31.3) in the on-demand, 4.9 (1.6–13.5) in the prophylaxis group, and 3.0 (2.0–6.8) in the prophylactic group of SHB. Three persons with SHA had zero bleeds during the observation period. On-demand therapy and hepatitis B and C were associated with higher ABR but not age, weight, and HIV positivity. Bleeding rates and the proportion of on-demand therapy in persons with hemophilia were high in our real-world cohort. Further improvement is needed, which might be facilitated with the advent of factor products with extended half-life or non-factor therapies.

**Electronic supplementary material:**

The online version of this article (10.1007/s00277-020-04250-9) contains supplementary material, which is available to authorized users.

## Introduction

Hemophilia, a rare bleeding disorder with X-chromosomal recessive inheritance pattern, is characterized by the deficiency of coagulation factor VIII (FVIII) in the case of hemophilia A or IX (FIX) in the case of hemophilia B. Severe hemophilia (defined as factor activity < 1%) is associated with a high risk of spontaneous bleeding, mostly affecting joints.

To prevent bleeding in severe hemophilia A and B (SHA and SHB), treatment with regular factor concentrate infusions is required [1–3]. In clinical practice, prophylaxis is frequently individualized based on bleeding phenotype, lifestyle and level of activity, pharmacokinetic profile, patient preference, and other factors [4]. Persons with severe hemophilia, who do not receive prophylactic factor replacement, are treated on demand, i.e., when bleeding occurs [2]. In high-income countries, an essential aspect of hemophilia management is home treatment [3].

The advantage of prophylaxis in reducing bleeding rates has been documented repeatedly in clinical trials. However, in everyday clinical practice, a huge variability in the management of hemophilia is reported [5, 6], and some studies suggested higher bleeding rates outside of clinical trials [5]. Despite the progress in developing new therapies, there could be a discrepancy between the clinical trial setting or guideline recommendations and treatment in a real-world setting [3, 4]. Therefore, we aimed at investigating the practice patterns of hemophilia treatment, bleeding rates, and the management of bleeding in a real-world cohort of persons with SHA and SHB, treated at a hemophilia center for adults in a Central European country between 2012 and 2017, a time period where most of the persons with hemophilia were receiving FVIII or FIX standard half-life concentrates.

## Materials and methods

### Study design, setting, and population

This was a retrospective single-center cohort study of persons with SHA and SHB to investigate the practice patterns of hemophilia treatment, bleeding frequency, and management of bleeding in a real-world setting. For this purpose, data from patient diaries, entries in the Austrian Hemophilia Registry [7–9], and medical records from the Hemophilia Center for adults at the Clinical Division of Hematology and Hemostaseology, Department of Medicine I, Medical University of Vienna, Austria, were evaluated between 01 January 2012 and 31 December 2017. At our center, persons with hemophilia are encouraged to record factor infusions and bleeding events requiring treatments in paper or electronic diaries and treatment protocols. From these sources, the following information was extracted: number and dates of infusions, type and amount (in international units (IU)) of factor product, intention of infusion (prophylactic or on-demand treatment), and nature (spontaneous, traumatic, or iatrogenic (e.g., surgery)) and location of bleeding. Furthermore, information on patients’ characteristics, comorbidities, and prescribed factor products were collected.

All male persons with SHA or SHB (factor VIII or IX baseline activity < 1%) aged ≥ 18 years were eligible. Subjects without available medical records and patient diaries were excluded. The detailed information (number and characteristics of all excluded subjects) is presented in Table A (supplementary file). A minimum documentation period of 6 months was required for study inclusion. We excluded patients with FVIII and FIX inhibitors (cutoff > 0.4 Bethesda Units (BU)) and those participating in interventional studies, until inhibitor eradication or completion of study participation (data prior to and after participation in interventional studies were evaluated). During the time period of this study, standard factor concentrates were the treatment of choice for hemophilia. Persons with factor concentrates with extended half-life were not eligible for this analysis.

The study was approved by the Ethics Committee of our institution (EK Nr: 1019/2018).

### Study objectives

The primary goal of the study was to describe a real-world cohort of persons with SHA and SHB without inhibitors with regard to treatment type and regimen and to investigate the annualized bleeding rate (ABR). Furthermore, factors correlating with the ABR were explored.

The secondary goal was to investigate the bleeding sites and bleeding management with regard to the number and dosage of factor concentrate infusions.

The tertiary goal was to describe the infusion intervals and probability of bleeding in subjects with prophylactic and on-demand treatment.

Definitions for treatment type, ABR, and target joints are described in Supplementary document 1.

### Statistical analysis

Categorical variables were presented as absolute frequencies and percentages; continuous variables as the arithmetic mean and standard deviation in the case of the normal distribution; and median and range or interquartile range (IQR, i.e., range between 25th percentile and 75th percentile) in the case of skewed distribution. The Kruskal-Wallis test was employed to compare non-normally distributed continuous variables between groups. Spearman’s rank correlation coefficient (*r*) was used to assess correlations between variables.

Univariate Poisson regression analysis was performed to test and quantify the effect of potential risk factors (type of hemophilia, hepatitis B and C, HIV positivity, age, and weight) on ABR. Furthermore, the influence of treatment (on demand vs. prophylactic) on the ABR was evaluated by a univariate Poisson regression model. To quantify the probability of bleeding, the cumulative incidence function was calculated, considering bleeding as a recurrent event and infusions without preceding bleeding as a competing event. Therefore, each day of infusion is regarded as an entry time point and time duration ends at the subsequent bleeding event or at the proximate infusion (= competing event), whichever occurs first. The cumulative incidences (= cumulative probabilities) of bleeding 1 week and 1 month after infusion are stated together with 95% confidence intervals (CI). The bootstrap percentile method was used for the calculation of CIs to allow for multiple observations per patient. To test for potential effects of the prognostic factors hepatitis B and C, HIV positivity, age, and weight on the bleeding probability, univariate Cox-regression models were applied on the recurrent event data, where infusions without preceding bleedings were considered as censored observations. To account for the inclusion of multiple observations per patient, the robust sandwich covariance estimate was calculated. Analyses of bleeding patterns were performed separately for the two treatment groups.

*p* values < 0.05 were considered as statistically significant. Statistical analyses were performed with SAS (version 9.4, SAS Institute Inc., 2016, Cary, NC, USA).

## Results

### Description of the study cohort

**Fig. 1:**
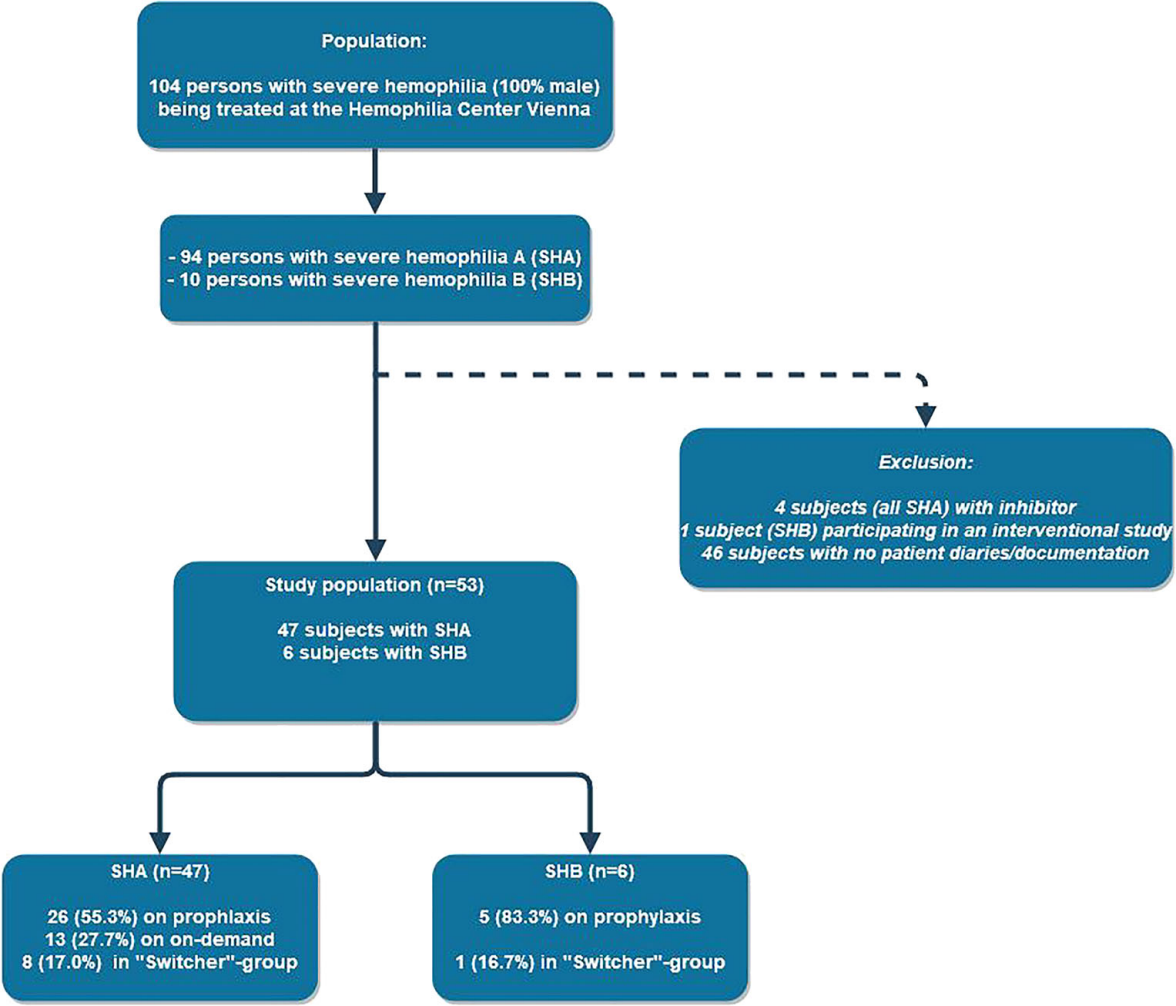
Total population of persons with severe hemophilia A and B during 2012 and 2017 and derivation of the study cohort

Ninety-four adult persons with SHA and 10 with SHB were treated at the Hemophilia Center Vienna during the observation period (01 January 2012 and 31 December 2017) (Fig. 1). Four subjects (all with SHA) were excluded because they had an inhibitor and one with SHB was excluded because he was participating in an interventional study over the entire observation period. Forty-six subjects (43 with SHA and 3 with SHB) could not be analyzed, as they did not have patient diaries or treatment protocols to extract data from. Therefore, the final study cohort consisted of 53 subjects, 47 with SHA (median age 38 (IQR 30–47) and 6 with SHB (median age 38 (IQR 28.3–58.3)).

The median observation time was 1096 days (IQR 744–1461) in subjects with SHA and 1374 days (IQR 365–1552) in those with SHB. The median infusion number (IQR) was 325 (114–471) in SHA and 294 (107–360) in SHB. Demographics, hemophilia-relevant information, and medical history are summarized in Table 1.

**Table 1:** Description of the study cohort and demographics

	Severe hemophilia A (*n* = 47)	Severe hemophilia B (*n* = 6)
Median (IQR) age, years	38 (30–47)	38 (28.3–58.3)
Median (IQR) height, cm	178.5 (173.8–181.0)	176.5 (167.8–180.3)
Median (IQR) weight, kg	79 (69–90)	72.5 (68.8–84.8)
Median (IQR) body mass index (BMI)	24.7 (2.6–28.1)	23.8 (22.1–27.2)
Mutation types, *n* (%)		
Inversion	19 (40.4)	-
Deletion	7 (14.9)	-
Missense	7 (14.9)	4 (66.7)
Nonsense	2 (4.3)	1 (16.7)
Splice site mutation	2 (4.3)	-
Outside of factor gene	1 (2.1)	-
Unknown	9 (19.1)	1 (16.7)
Arthropathy, *n* (%)	36 (76.7)	6 (100)
Knee joints, *n* (%)	25 (53.2)	3 (50)
Ankle joints, *n* (%)	20 (42.6)	4 (66.7)
Elbow joints, *n* (%)	22 (46.8)	0 (0)
Shoulder joints, *n* (%)	3 (6.4)	0 (0)
Hip joints, *n* (%)	2 (4.3)	0 (0)
Unknown joint status, *n* (%)	4 (8.5)	0 (0)
HIV positive, *n* (%)	6 (12.8)	1 (16.7)
History of hepatitis B, *n* (%)		
Positive	2 (4.3)	0 (0)
Negative	21 (66.0)	5 (0)
SVR*/spontaneous resolution	14 (29.8)	1 (16.7)
History of hepatitis C, *n* (%)		
Positive	17 (36.2)	2 (33.7)
Negative	18 (38.3)	4 (66.7)
SVR*/spontaneous resolution	12 (25.5)	0 (0)
Median (IQR) observation time (i.e., analyzed time frame), days	1096 (744–1461)	1374 (365–1552)
Total number of infusions (i.e., exposure days) during the observation period in all patients	15,453	1600
Median (IQR) number of infusions (i.e., exposure days) during observation per patient	325 (114–471)	294 (107–360)
Total exclusion time, days*	3229	0

Overall findings, demographics, mutations, and hemophilia-associated comorbidities. *IQR*, interquartile range; *HIV*, human immunodeficiency virus; *SVR*, sustained virologic response

*Exclusion time includes time periods where subjects participated in an interventional study and time periods where subjects did not provide documentation on their treatment

### Treatment patterns and factor consumption

Twenty-six (55.3%) persons with SHA were on prophylaxis, 13 (27.7%) on on-demand treatment, and 8 (17.0%) were assigned to the “switcher group.” In the “switcher group,” 7 subjects switched from on-demand treatment to prophylaxis and 1 subject from a prophylactic regimen to on-demand therapy. Five (83.3%) persons with SHB were on prophylaxis and 1 (16.7%) was assigned to the “switcher group,” switching from on-demand treatment to prophylaxis.

Table 2 summarizes product and treatment types, and factor utilization. Median prescribed (IQR) international unit per kilogram per week for persons with SHA and SHB on prophylaxis was 66.7 (53.8–87.0) and 40.1 (30.9–57.3), respectively. Most persons with SHA performed the prophylactic treatment with 3 infusions per week and the majority of SHB subjects with 2 infusions per week. Median (IQR) factor consumption was 2290 (1020–3449) and 1808 (1627–3047) IU/kg/year for all subjects with SHA and SHB, respectively.

**Table 2:** Treatment patterns and factor utilization in patients with severe hemophilia A and B

	Severe hemophilia A (*n* = 47)	Severe hemophilia B (*n* = 6)
Treatment regimen		
Prophylaxis, *n* (%)	26 (55.3)	5 (83.3)
On demand, *n* (%)	13 (27.7)	0 (0)
“Switcher group”, *n* (%)	8 (17.0)	1 (16.7)
Factor product type, *n* (%)		
Recombinant	35 (74.5)	2 (33.3)
Plasma derived	12 (25.5)	4 (66.7)
Prescribed factor dosage in prophylaxis	*n* = 31*	*n* = 6
Median (IQR) IU/kg/week	66.7 (53.3–87.0)	40.1 (30.9–57.3)
Mean (± SD) IU/kg/week	71.7 (± 23.4)	42.5 (± 12.9)
Prescribed weekly infusions, *n* (%)	*n =* 31*	*n* = 6
1/week	1 (3.2)	0 (0)
1–2/week	2 (6.4)	1 (16.7)
2/week	3 (9.7)	4 (66.7)
2–3/week	4 (12.9)	1 (16.7)
3/week	13 (41.9)	0 (0)
3–4/week	8 (25.8)	0 (0)
Median (IQR) factor consumption (IU/year)	190,051 (89,250–254,892)	143,655 (109,662–223,353)
Median (IQR) factor consumption (IU/kg/year, all subjects)	2290 (1020–3449)	1808 (1627–3047)
Prophylaxis	3364 (2219–4297)	1791 (1482–3063)
On demand	921 (461–1275)	-
“Switcher”	2382 (1971–2838)	1826

Practice patterns among the study cohort regarding treatment regimen, product type, prescribed factor dosing, and frequency of prophylaxis and consumed factor concentration

*For prescribed factor dosage in the prophylaxis group, *n* is lower as this value could only be calculated for subjects who were on prophylaxis at some point in the observation period. This includes all subjects with SHA on prophylaxis and 5 subjects from the “Switcher” group who were prescribed a defined prophylactic regimen. For 3 subjects of the “switcher group” with SHA, prescribed weekly prophylactic infusions were unknown

### Rates, types, and locations of bleeding

Table 3 shows the total number of bleeding events and ABR (all bleedings and joint bleeds only) according to the treatment regimen.

**Table 3:** Bleeding outcomes (total bleeding events, annualized bleeding rates (ABR), and joint bleeding) according to the treatment regimen

	Severe hemophilia A (*n* = 47)	Severe hemophilia B (*n* = 6)
All recorded bleeding events during observation, *n*	2715	102
Recorded bleeding events per subject during observation, median (IQR)	36.0 (12–82)	9.5 (1.8–37)
Spontaneous, median (IQR)	29 (8–65)	5.5 (0.8–28.5)
Traumatic, median (IQR)	2 (0–6)	4.5 (0–7.5)
Unknown cause, median (IQR)	0 (0–2)	0 (0–0.3)
ABR (all bleeding events) according to the treatment regimen, median (IQR)		
Total	13.8 (4.5–24.9)	4.9 (1.6–8.5)
Prophylaxis	4.9 (1.6–13.5)	3.0 (2.0–6.8)
On demand	28.0 (23.4–31.3)	-
“Switcher group”	18.4 (12.7–23.4)	13.1 (-)
Subjects with ABRs = 0, *n* (%)	3 (6.4)	0 (0)
Subjects with ABRs > 0 and ≤ 3, *n* (%)	4 (8.5)	3 (50.0)
Subjects with ABRs > 3 and ≤ 6, *n* (%)	9 (19.1)	0 (0)
Subjects with ABRs > 6, *n* (%)	31 (66.0)	3 (50.0)
ABR (joint bleeding) according to the treatment regimen, median (IQR)		
Total	9.8 (3.4–19.1)	2.1 (0.8–6.4)
Prophylaxis	4.1 (0.9–12.3)	2.0 (0.5–3.8)
On demand	21.6 (12.5–28.8)	-
“Switcher group”	13.2 (9.4–18.5)	9.4 (-)

*ABR*, annualized bleeding rate

In SHA, the highest ABR was observed in subjects with on-demand treatment, followed by the “switcher group,” and persons with a prophylactic regimen had the lowest ABR (median ABR, 28.0 vs. 18.4 vs. 4.9, *p* < 0.001). Two persons with SHA on prophylaxis had a very high ABR. Their clinical characteristics are described in Supplementary document 2. Only 3 persons with SHA had zero bleeds during the entire observation period. In persons with SHB, statistical comparisons between treatment regimens could not be performed due to low numbers.

In persons with SHA, the median (IQR) ABR for spontaneous bleeds only was 4.1 (1.0–12.3) in the prophylaxis, 22.0 (17.5–32.5) in the on-demand and 15.2 (10.5–20.4) in the “switcher” group. For subjects with SHB, the median (IQR) ABR for spontaneous bleeds was 2.0 (0.5–3.6) for the prophylaxis and 11.1 (-) for the “switcher” group. The bleeding sites are summarized in Table 4.

**Table 4:** Annualized bleeding rate (ABR) according to sites of bleeding and treatment regimen, and number of target joints

	Severe hemophilia A (*n* = 47)			Severe hemophilia B (*n* = 6)		
ABR according to sites of bleeding, median (range)	Total			Total		
Joint	9.8 (0–82.5)			2.1 (0–9.4)		
Muscle/CT	1.3 (0–10)			0.4 (0–3.1)		
Dental/gingival	0 (0–3.2)			0 (0–0.3)		
Gastro-intestinal	0 (0–2.6)			0 (0–0.3)		
Injury	0 (0–15.5)			0 (0–0)		
Other	0.5 (0–5.7)			0.3 (0–6)		
ABR according to the site in different treatment regimens, median (range)	Prophylaxis	On demand	Switcher	Prophylaxis	On demand	Switcher
Joint	4.1 (0–82.5)	21.6 (6–48)	13.2 (5.6–22.3)	2 (0–5.4)	-	9.4 (-)
Muscle/CT	0.1 (0–8.5)	3.8 (1–10)	2.7 (0–4.3)	0 (0–1.4)	-	3.1 (-)
Dental/gingival	0 (0–3.2)	0 (0–2.8)	0.4 (0–1.7)	0 (0-1.4)	-	0 (-)
Gastro-intestinal	0 (0–1)	0 (0–0.5)	0.1 (0–2.6)	0 (0–0)	-	0.3 (-)
Injury	0 (0–15.5)	0 (0–8.7)	0.8 (0–2.5)	0 (0–0)	-	0 (-)
Other	0.2 (0–3.8)	0.6 (0–5.7)	0.7 (0–1.2)	0.3 (0–6)	-	0.3 (-)
Number of target joints, median (IQR)	1 (0–5)	4 (0–9)	2 (0–5)	0 (0–2)	-	1 (-)

Bleeding sites were classified as joint (ankle, knee, hip, wrist, elbow, and shoulder joint bleedings), muscle and connective tissue (CT, as specified by subjects), dental/gingival (gum bleeds, dental bleeding events), gastro-intestinal (all bleeding events affecting the gastro-intestinal tract, including the occurrence of melena), injury (traumatic bleeding events not affecting joint or muscle and not including surgeries, e.g., skin bleeds from shaving, hematomas, tongue bites), other (surgeries including joint and general surgery and bleeding events occurring at a very low frequency, e.g., epistaxis, ocular bleeds, hematuria, and all unknown bleeding sites)

### Correlations and association with bleeding events

There was a strong correlation between the number of target joints and ABR (*r* = 0.80, *p* < 0.001). There was no correlation between ABR and body weight (*r* = − 0.01, *p* = 0.921), the correlation of ABR with age (*r* = 0.36, *p* = 0.007), or factor consumption per year (*r* = − 0.36, *p* = 0.008) was weak-to-moderate.

With the Poisson regression analysis, we analyzed the influence of patient characteristics on ABR. There was a statistically significant influence of hepatitis B and C on ABR (3.3-fold increase in persons with a history of hepatitis B and C, 95% CI 1.62–6.82, *p* = 0.001). As expected, there was a significant difference between on-demand and prophylactic treatment (a 2.8-fold increase of ABR with on-demand treatment, 95% CI 1.65–4.84, *p* < 0.001). There was no significant influence of age (1.02-fold increase, 95% CI 1.00–1.04, *p* = 0.076) and weight (0.96-fold, 95% CI 0.98–1.02, *p* = 0.725) on ABR. Also, no statistically significant effect of type of hemophilia (3.5-fold increase in SHA vs. SHB, 95% CI 0.88–13.7, *p* = 0.076) or HIV positivity (1.40-fold increase, 95% CI 0.74–2.64, *p* = 0.302) on ABR was found.

### Bleeding management

In the total study cohort, the median number of infusions per bleeding event was 1 (range 1–6) and the median (IQR) treatment dosage per bleeding event was 3026 (2063–4000) IU factor concentrate. The median dosage (IQR) per bleeding event was 3550 (2752–4584) IU factor concentrate in the prophylaxis, 2115 (1538–2500) IU in the on-demand and 2076 (1791–4717) IU in the “switcher” group. The median (IQR) number of maximum infusions for treating a bleeding event was 5.0 (2.5–9.5) in the prophylaxis, 4.0 (3.0–6.0) in the on-demand, and 4.0 (2–12) in the “switcher” group.

### Infusion intervals, probability of and factors associated with bleeding

In subjects with SHA, the median (IQR) infusion interval was 2.6 (2.1–3.5) days in the prophylaxis, 11.1 (6.5–12.3) days in the on-demand, and 3.8 (3.2–5.3) days in the “switcher” group. In subjects with SHB, the median (IQR) infusion interval was 3.8 (3.4–4.9) days in the prophylaxis and 4.2 (-) in the “switcher” group.

**Fig. 2:**
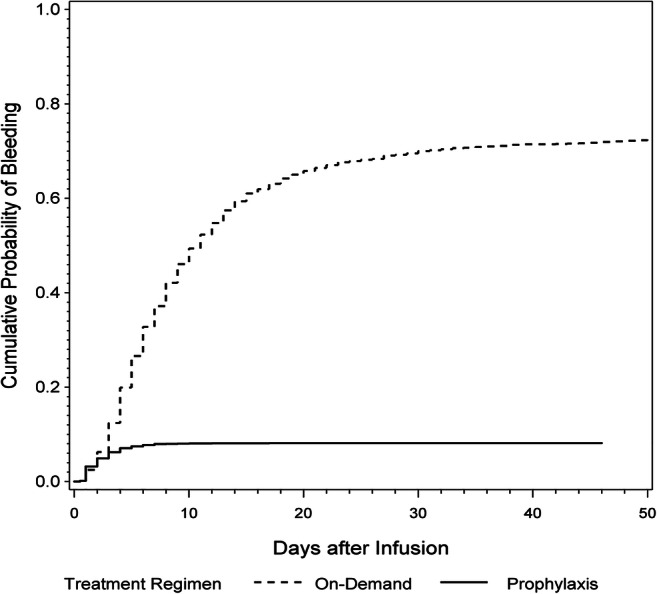
The cumulative probability of bleeding in all persons with severe hemophilia A and B in the prophylactic and on-demand treatment group. The curves depict the estimates of the cumulative probability of bleeding after infusion in the two treatment regimens. Every single infusion episode is used for calculation and each bleeding event is represented by a step in the curve. Due to the different treatment strategies, the timing of the bleeding events differs substantially and the flattening of the prophylaxis treatment curve after 7 days reflects the fact that patients in this group usually apply an infusion at least once a week. The number of observations (= number of infusion episodes) at risk at days 0/5/10/15/20/25/30 is 1693/960/483/251/148/92/63 in the on-demand group; 12647/1084/91/30/16/6/4 in the prophylaxis group

To analyze bleeding patterns, we estimated the bleeding probabilities by cumulative incidence functions (considering infusions without preceding bleeding as competing events) in subjects with SHA and SHB on prophylactic and on-demand treatment (Fig. 2).

In the prophylaxis group, the probability of observing a bleeding event within the first week after the infusion was 8.0% (95% CI 3.4%–13.8%) and within the first month 8.1% (95% CI 3.5%–14.0%). History of hepatitis B or C was the only significant predictor for a shorter time to next bleeding event (hazard ratio (HR), 95% CI 4.53, 1.82–11.27, *p* = 0.001). Age (1.03, 0.98–1.08, *p* = 0.194), weight (0.96, 0.90–1.03, *p* = 0.283), and HIV (2.78, 0.62–12.53, *p* value 0.183) were not associated with time to next bleeding.

In the on-demand group, the bleeding probability within the first week after the infusion was 37.2% (95% CI 23.2%–47.9%) and increased to 70.0% (95% CI 59.7%–78.0%) after one month; Fig. 2. Hepatitis B and C (HR, 95% CI 1.79, 1.39–2.30, *p* < 0.001) and HIV (1.78, 1.24–2.56, *p* = 0.002) were significantly associated with a shorter time to bleeding, while higher weight (0.99, 0.98–1.00, *p* = 0.030) was significantly associated with a longer time to bleeding. Age (0.99, 0.98–1.00, *p* = 0.065) was not associated with time to bleeding.

## Discussion

We analyzed treatment patterns and described bleeding outcomes, management of bleeding, and bleeding probability in clinical practice in persons with SHA and SHB, covering a time period before the introduction of new hemophilia treatments with extended half-life factor products and non-factor therapies. According to the latest update of the Austrian Hemophilia Registry, which covers more than 85% of the assumed persons with hemophilia in Austria, there are 294 persons with SHA or SHB in Austria. Of those, 53 (18.0%) were included in our study, which has been performed at a single center [9].

While real-world data are available for many high-income countries, no such analysis has yet been performed in Austria [5, 6, 10]. The majority of persons with SHA (55.3%) and SHB (83.3%) were on prophylaxis. The high proportion of prophylaxis is expected as it is the preferred treatment recommendation in national and international guidelines [2, 3]. In a recent real-life cohort study, which analyzed treatments in several high-income European countries, a prophylaxis rate between 50% (e.g., in France) and almost 100% (e.g., in Sweden) was reported in SHA and a prophylaxis rate from 25–100% in SHB [5]. The prophylaxis rate in our cohort is within this range. Despite availability and access to factor concentrates, the main reasons for rejecting prophylaxis could be difficult venous access to perform regular i.v. injections or patient preference to favor on-demand therapy. However, we were not able to define the exact cause of not performing prophylaxis in this retrospective analysis.

Most persons with SHA and SHB were prescribed 3 and 2 infusions per week, respectively, as suggested in the Austrian consensus report for hemophilia treatment [2]. The mean prescribed dosage for prophylaxis in SHA in our cohort (71.7 IU/kg/week) was lower than in other high-income European countries such as Sweden, Germany, Italy, Spain, France, and the UK (ranging from 88.5–108.4 IU/kg/week) and Australia (84.4 IU/kg/week), however, similar to that in Belgium (69.0 IU/kg/week). Only in the Netherlands, where a low-dose prophylaxis regimen is followed (15–30 IU/infusion 2–3 times per week), the prescribed median (mean values not reported) factor dosage was less than in Austria (46.0 versus 66.7 IU/kg/week) [11, 12]. In our SHB cohort, the mean prescribed factor (42.5 IU/kg/week) was comparable comparable with Germany (41.4 IU/kg/week) but lower than in most high-income European countries (ranging from 63.6-97.7 IU/kg/week) [5, 6].

Our study provides data on bleeding outcomes, expressed as ABR, in persons treated with standard half-life products in a real-world setting. The median ABR in subjects with SHA and SHB on prophylaxis was 4.9 and 3.0, respectively. These numbers are comparable with those in other high-income countries such as France (4.0) and the UK (4.0) but slightly higher than in Germany (2.0), Spain (2.0), Sweden (1.0), Belgium (1.0), and Italy (1.0). Interestingly, Belgium, which follows a low-dose prophylactic regimen similar to Austria, however, achieves a median ABR as low as 1.0, suggesting the possibility of a decreased prophylactic dose without increasing the bleeding rate [5].

It was difficult to compare our real-world data with prospective cohort studies as demographics and methods of data collection differed strongly. However, one such study showed a slightly lower median ABR for subjects with SHA on prophylaxis (2.6), despite the fact that it included also subjects with moderate hemophilia [13]. Three out of 53 (5.7%) subjects, all of whom had SHA and were on prophylaxis, achieved zero bleeds. This is also observed in other high-income countries, where the percentage of persons with zero bleeds ranges from 0–41.7% [5]. Generally, this percentage strongly depends on the observation time as the likelihood of experiencing a bleed increases the longer a subject is observed.

We also investigated factors associated with ABR and found only a significant association of higher ABRs with active or history of hepatitis B and C. Thus, prophylaxis and strict adherence to the prophylactic treatment regimen are essential for this subgroup. Additionally, persons with severe hemophilia and active infections should be strongly advised to undergo effective antiviral treatments for chronic hepatitis C to avoid disease-related complications [14].

The most common bleeding sites were joints, and the number of target joints was higher in the on-demand group. This was expected, as hemarthrosis is the hallmark of hemophilia [1].

When calculating factor consumption in the prophylaxis group, we found that prescribed dosage and mean annual consumption for both SHA (3485 IU/kg/year) and SHB (2176 IU/kg/year) was lower compared with other high-income countries (ranging from 3588–5636 IU/kg/year) [5, 6]. We also analyzed the bleeding management in our cohort. The median dosage infused per bleeding event differed across treatment groups: it was highest in the prophylactic (3550 IU), followed by the on-demand (2115 IU) and the “switcher” group (2076 IU). Similar data on bleeding management and factor use is scarce. In one prospective study, one infusion of 2000 IU was enough to treat most bleeds [13].

Furthermore, we estimated the bleeding probability for subjects on prophylaxis and on-demand treatment and found, as expected, a higher probability of bleeding in the latter (8.0% vs. 37.2% within 1 week of observation). Persons with on-demand therapy would benefit from the initiation of prophylaxis to significantly decrease the bleeding probability. The analysis of the bleeding probability and time to bleeding was a new concept to illustrate differences between prophylactic and on-demand treatment and the factors influencing the bleeding risk.

Our study has several limitations. In Austria, it is not mandatory to keep diaries and track infusions and bleeding events. Thus, selection bias is likely. As the study is retrospective, using patient diaries, which were kept by subjects themselves, the completeness and accuracy of our data heavily relied on the subjects’ diligence. To counteract this issue, we excluded time frames in which subjects did not make entries for longer time periods in spite of a previously impeccable prophylactic regimen. Also, the time frame over which patient diaries were kept varied between subjects, ranging from 191–1827 days. Another limitation was the low number of subjects, especially with SHB, which made it difficult to draw strong conclusions. The fact that we had to introduce a third treatment group for subjects, who switched from on-demand to prophylaxis treatment or vice versa, further reduced the patient number assigned to one of the two groups. However, we believe that this was necessary, as subjects in the “switcher” group differed from those in the prophylactic group.

In summary, bleeding rates in persons with severe hemophilia treated with standard half-life concentrates were high in our real-world cohort, as was the proportion of on-demand therapy in those with SHA. Increasing the number of persons on prophylaxis and establishing individualized treatment concepts, especially for those with a history of hepatitis, may further reduce ABRs and improve joint function and quality of life. In addition to currently available therapies, the availability of new factor products with extended half-life or non-factor therapies could potentially lead to further improvements.

## Electronic supplementary materials

ESM 1 (DOCX 91 kb)

ESM 2 (DOCX 86 kb)
