# Supplementary material for: Treatment patterns and bleeding outcomes in persons with severe hemophilia A and B in a real-world setting
Source: Ann Hematol. 2020 Sep 11;99(12):2763–71. doi: 10.1007/s00277-020-04250-9 (PMC7683481; doi:10.1007/s00277-020-04250-9)
Supplement: Supplementary file 1 — (DOCX 91 kb) [file 277_2020_4250_MOESM1_ESM.docx]

**Definitions (types of treatment, ABR and target joints)**

For hemophilia A and B as well as hemophilia-associated/non-hemophilia-associated co-morbidities, we relied on data from medical records including medical history and diagnostic codes assigned by treating physicians. Practice patterns and bleeding events were documented by persons with hemophilia in their patient diaries, in the Austrian Hemophilia Registry or in medical records of the Hemophilia Center Vienna, Vienna, Austria.
Prophylactic treatment (or prophylaxis) of severe hemophilia was defined as the regular intravenous replacement of factor concentrate (VIII or IX) at least once a week to prevent spontaneous bleedings, according to the Austrian Hemophilia Consensus Report.^[2]^ The dosage and frequency of application had to be defined before initiating prophylactic treatment. On-demand treatment was defined as having no prescribed prophylactic treatment.^[7,8]^ A few subjects switched from on-demand treatment to prophylactic or vice versa during the time period of the study, and these subjects were categorized in a separate group, referred to as “switcher group”.
The ABR was defined as the number of bleeding events within 12 months.^[7]^ If a subject had a shorter observation period than 12 months, the available data was extrapolated to allow the calculation of the ABR.
Target joints were defined as joints in which 3 or more spontaneous bleeds had occurred over a time period of 6 consecutive months.^[5]^
