# Supplementary material for: Treatment patterns and bleeding outcomes in persons with severe hemophilia A and B in a real-world setting
Source: Ann Hematol. 2020 Sep 11;99(12):2763–71. doi: 10.1007/s00277-020-04250-9 (PMC7683481; doi:10.1007/s00277-020-04250-9)
Supplement: Supplementary file 2 — (DOCX 86 kb) [file 277_2020_4250_MOESM2_ESM.docx]

We gathered data on excluded subjects who had no patient diaries and documentation of their treatment and bleeding events . There were 43 (93.5%) subjects with severe hemophilia A (SHA) and 3 (6.5%) with severe hemophilia B (SHB). Interestingly, in subjects with SHA, there were more subjects on on-demand treatment than on prophylaxis. Furthermore, among subjects with SHA that could not be included due to missing patients’ diaries, a large proportion was HCV positive (44.2%).

**Table A: Characteristics of patients without patient diaries**

|  | Severe  Hemophilia A (n=43) | Severe  Hemophilia B (n=3) |
| --- | --- | --- |
| Treatment regimen, n (%) |  |  |
| *Prophylaxis* | *20 (46.5)* | *3 (100)* |
| *On-Demand* | *23 (53.5)* | *0 (0)* |
| Median (IQR) age, years | 37 (28-50) | 29.0 (21.0-n/a) |
| Median (IQR) height, cm* | 178.0 (170.0-182.3) | 180.0 (175.0-n/a) |
| Median (IQR) weight, kg* | 75.0 (68.5-88.0) | 75.0 (73.0-n/a) |
| Median (IQR) body mass index (BMI)* | 24.9 (21.5-28.0) | 23.1 (20.8-n/a) |
| Mutation types, n (%) |  |  |
| *Inversion* | *17 (39.5)* | *-* |
| *Deletion* | *2 (4.7)* | *-* |
| *Missense* | *7 (16.3)* | *1 (33.3)* |
| *Nonsense* | *2 (4.7)* | *1 (33.3)* |
| *Splice site mutation* | *2 (4.7)* | *-* |
| *Unknown* | *13 (30.2)* | *1 (33.3)* |
| Arthropathy, n (%) | 33 (76.7) | 2 (66.7) |
| *knee joints, n (%)* | *13 (30.2)* | *-* |
| *ankle joints, n (%)* | *15 (34.9)* | *1 (33.3)* |
| *elbow joints, n (%)* | *11 (25.6)* | *1 (33.3)* |
| *shoulder joints, n (%)* | *4 (9.3)* | *-* |
| *hip joints, n (%)* | *2 (4.7)* | *-* |
| *Unknown joint status, n (%)* | *7 (16.3)* | *1 (33.3)* |
| *HIV positive, n (%)* | *10 (23.3)* | *0 (0)* |
| *History of Hepatitis B, n (%)* |  |  |
| *positive* | *-* | *-* |
| *negative* | *28 (65.1)* | *2 (66.7)* |
| *SVR*/spontaneous resolution* | *14 (32.6)* | *1 (33.3)* |
| *unknown* | *1 (2.3)* |  |
| *History of Hepatitis C, n (%)* |  |  |
| *positive* | *19 (44.2)* | *1 (33.3)* |
| *negative* | *18 (41.9)* | *2 (66.7)* |
| *SVR*/spontaneous resolution* | *5 (11.6)* | *-* |
| *unknown* | *1 (2.3)* | *-* |
